# Grand Challenge in Sleep and Sleep Medicine: The Road Ahead

**DOI:** 10.3389/frsle.2022.919642

**Published:** 2022-06-21

**Authors:** Stuart F. Quan

**Affiliations:** ^1^Division of Sleep and Circadian Disorders, Brigham and Women's Hospital and Harvard Medical School, Boston, MA, United States; ^2^Asthma and Airway Disease Research Center, University of Arizona College of Medicine, Tucson, AZ, United States

**Keywords:** sleep, insomnia, circadian disorders, obstructive sleep apnea, precision sleep medicine, mental health

“Sleep and watchfulness, both of them, when immoderate, constitute disease” (Hippocrates, 400 B.C.E.). This statement by the ancient Greek physician Hippocrates is evidence that early civilizations recognized the importance of healthy sleep in their lives. However, beyond empiric observations, there was little in the way of formal sleep research until the 20^th^ century in part because of the lack of a means to objectively assess sleep. The invention of the electroencephalograph in 1875 by Caton changed this landscape (Caton, 1875). Subsequently, the seminal studies describing the human EEG by Berger in 1929 (Berger, 1929) and sleep stages by Loomis and colleagues in 1937 (Loomis et al., 1937) ushered in the modern era of sleep research.

It is now 85 years after those seminal studies, research into sleep, and sleep and circadian disorders has increased exponentially. In the decade from 1960 to1969, there were 2,221 citations in PubMed containing “sleep” in their title. In comparison, from 2010 to 2019, this had grown to 43,222, a 19.5-fold increase. Correspondingly, this has led to significant advances in our understanding of the nature and importance of sleep which in turn has translated into the ability to recognize and treat over 100 sleep disorders (Sleep Disorders, 2022). These advances include the Nobel Prize winning discovery of the molecular mechanisms that control circadian rhythms and hence the daily cycle of sleep and wakefulness (The Nobel Prize, 2017), the importance of sleep in processing and retaining information (Miletínová and Bušková, 2021), the negative impact of sleep deficiency on metabolic and cardiovascular health (Miletínová and Bušková, 2021), and the ability to diagnose and treat common sleep disorders such as obstructive sleep apnea (OSA) and insomnia (Pavlova and Latreille, 2019). Although these and other important discoveries have markedly advanced the fields of sleep and circadian disorders, there is much more to learn and understand.

In basic and translational sleep research, there are several areas that require more investigation. For example, the roles of genomics, proteomics and microbiomics in understanding the physiology of sleep and their application to diagnosing and treating sleep disorders need to be determined (O'Callaghan et al., 2019). The current epidemic of COVID-19 and its association with poorer sleep quality, insomnia (Cénat et al., 2021) and OSA (Hariyanto and Kurniawan, 2021) is a reminder that linkages between sleep and immune function (Liu et al., 2021) are another area that requires more clarity. Although there is some evidence that better sleep improves immunity to infection (Besedovsky et al., 2012), more insight into the underlying mechanisms and how this knowledge can be applied to real applications is needed. With our current understanding of the mechanisms underlying circadian rhythms, can this be applied therapeutically to treat those who are shift-workers, currently as many as 20% of the population? Furthermore, can this knowledge be applied to treat those with other circadian rhythm disorders or other chronic diseases? There is pressing need for the development of biomarkers to both identify sleep disorders and track their progress (Quan, 2011). Increasing knowledge in these areas and others are the tasks to be accomplished by basic and translational investigators.

In the clinical arena, new therapeutic approaches are being rapidly developed. However, an important deficiency for many of those who develop these treatments is the lack of the requisite rigorous testing to recommend their clinical application. With respect to some of the major sleep disorders, there are numerous areas that require clinical investigation. For OSA, diagnosis is rapidly migrating to home sleep testing (HST), but HST's still incur significant cost. Is there a biomarker which could be used in conjunction with other screening instruments to identify those with OSA? Continuous positive airway pressure (CPAP) is the most effective although not necessarily the most efficacious treatment for OSA, but it is prescribed for most individuals. Oral appliances also are prescribed for those who are unable or unwilling to use CPAP (Pavlova and Latreille, 2019). However, there are few other cost-effective treatment alternatives. A major obstacle in this area is the lack of broadly applicable novel therapeutic modalities for OSA. For insomnia, cognitive behavioral therapy (CBT-i) is recognized as the best initial treatment (Qaseem et al., 2016), but availability is limited by the number of qualified clinicians. Can access be improved through online delivery? New hypnotics are being developed. What is their role in insomnia treatment? Is long-term use a risk factor for adverse outcomes such as dementia? (Ettcheto et al., 2020).

There are many additional unanswered questions for OSA, insomnia and other sleep disorders as well. However, one broadly applicable goal for all sleep disorders is to define a role for Precision Medicine (Patil, 2019). Tailoring treatment to a specific genotype or phenotype to deliver cost-efficient care is an emerging area in Sleep Medicine. The initial obstacle will be to identify specific genotypes or phenotypes that might differentially respond to treatment. A more difficult hurdle will be to develop targeted therapies for them.

Perhaps the greatest and most ambitious goal for the sleep field is to imbue a culture of healthy sleep practices into the world's societies. At least 7 h of sleep per night is recommended for optimum health, yet in developed countries, a large percentage of adults fail to achieve this threshold (Chattu et al., 2018). Factors contributing to this high level of sleep deficiency include impact of artificial lighting on circadian rhythms, the needs of a 24 h society and an “always on” work ethic. However, there is increasing evidence that sleep deficiency contributes to adverse outcomes for many chronic diseases such as diabetes, obesity, and cardiovascular disease (Chattu et al., 2018). A change in societal attitude can only be accomplished through the joint efforts of government, private institutions and concerned individuals backed by scientific data and advocacy efforts from sleep clinicians and scientists.

Are we ready to move forward to close the many knowledge gaps in the field of sleep and circadian disorders? As clinicians and scientists in our field let us challenge ourselves to do so and to make the world a place with healthy sleep. The road ahead is replete with the wonders of discovery and the gratification of accomplishment.

## Author Contributions

The author confirms being the sole contributor of this work and has approved it for publication.

## Conflict of Interest

SQ is a consultant for Whispersom, DR Capital, Bryte Bed, and Best Doctors.

## Publisher's Note

All claims expressed in this article are solely those of the authors and do not necessarily represent those of their affiliated organizations, or those of the publisher, the editors and the reviewers. Any product that may be evaluated in this article, or claim that may be made by its manufacturer, is not guaranteed or endorsed by the publisher.
